# Supplementary material for: Prevalence of low birth weight and its associated factor at birth in Sub-Saharan Africa: A generalized linear mixed model
Source: PLoS One. 2021 Mar 11;16(3):e0248417. doi: 10.1371/journal.pone.0248417 (PMC7951905; doi:10.1371/journal.pone.0248417)
Supplement: Attachment — Submitted filename: Reviewer_comments.pdf [file pone.0248417.s002.pdf]

The topic of determinants of low birth weight at birth is of high interest, particularly in sub-Saharan Africa. The manuscript is clearly written and presented.

However, there are some aspects that the authors should consider.

Major comments

1. The principal issue of this work is the use of DHS data.

DHS is known as a formidable source of data and give a general overview of sanitary situation of each country (trend of key indicators). However, there are many methodological concerns about accuracy of data collection, population sample and high risk of potential biases: Selection biases (due to different household sampling techniques), information or classification biases (due to self-report data or declared data, medical records, standardization of data collection …). In addition, the methodology can differ between countries, especially concerning the under-five children data. Indeed, two main different methods can be used to collect these data: Most-Recently-Born Children and Non-Most-Recently-Born Children.

I invite the authors to read this critical analysis of the use of DHS data for children (pdf attached), whose conclusion I quote: "When researchers use the DHS surveys for the analysis of children born in the five years preceding the survey, they must be very careful to consider and avoid inherent biases that derive from both the data structure and the nature of the analysis. Beyond the usual considerations of omission of births and transference of births across questionnaire age boundaries, particularly the health section and calendar age/date boundaries, using data for last-born children or non-last-born children instead of all children born in the five years preceding the survey will probably result in biased research findings."

2. Analysis (statistical issue)

Multiple pregnancies (such as twin pregnancy) were included in the same models with single pregnancy. Multiple pregnancies (MP) are known as a mediator factor of LBW (MP is on the pathway of LBW). Therefore, this can lead to a sur-adjustment. I will be more useful to perform the main analysis after excluding MP and to perform a sensitivity analysis with MP.

Other comments

The use of "Determinants" in the title by the authors is misleading to me. The notion of determinant implies a causal role while the notion of risk factor or associated factor is broader and refers to a higher probability of disease in exposed subjects. In this work, it is rather "associated factors" that have been highlighted.

Abstract

Lines 47-48: In conclusion of abstract, the authors declared "this study… with significant variations among countries" but any variations or range have been presented in the results paragraph of abstract. Please, correct it.

Introduction

Lines 71-72: 25-30 times seems very high today (substantial progress has been made, even in countries with limited resources). I suggest that the authors further clarify this information,

either by adding that this very high risk is for very low birth weight infants, or that the information is several years old.

Lines 80-82: There are many studies such as systematic reviews, multicenter pooled analyses (some of which have even been cited by the authors). The rationale for this study therefore needs to be better specified. For the moment, we do not really see the added value or the new knowledge brought.

Lines 85-86: I am not convinced.

Methods

See my major comment about the use of DHS data. I suggest that the authors briefly present the methods used to collect data on children in the DHS to give us an idea of the robustness or otherwise of the data and potential biases.

Also, what about children who were not included in this analysis. Do the authors check any potential selection biases?

It will be useful to have a country included in each region.

How infant weight were measured at birth? Standardization? Declared (or self-report data?) Medical record?

Variable definition paragraph is missing in this work, particularly for the clarification of the definition of variable such as "household wealth index", "women health care decision making autonomy".

What about other important independent variables such as malaria infection or BMI?

ANC: please, define the acronym the first time you use it.

The parameters of mixed logistic regression need to be more detail. Indeed, the authors used multilevel models to take into account the hierarchical structure of data. However, it is important to specify what represents the 1st level (individual?), the 2nd level (regions or countries?). Please, clarify.

Results

Table 2: West Africa weighted frequency is 12170, but Benin (which is West African country) only provide 13909 participants. Please, clarify.

Table 4: It will be useful to have also percentage in normal and LBW columns.

Lines 162-163: "Women from a cluster … lower birth weight". This sentence is a bit confusing. Please, clarify.

Discussion

Line 197: "8.9% to 10.3%", please include this result in results session also.

In general, the discussion section need to be more focus on comparison of this study with other multicentric pooled analysis and meta-analysis studies in sub-Saharan Africa, instead of comparison of single center or no-pooled analysis studies.

Lines 199-207: For me, these comparisons are not relevant (single center studies vs. pooled multicenter study).

Lines 214-224: All this paragraph is a speculation. What about nutritional factors in this study. Iron supplementation was not associated with LBW in this study. Why? A discussion of this negative finding will be useful. Why didn't the authors consider other indicators of nutritional status such as BMI? If nutritional status plays an important role in the occurrence of LBW, all of the above should be discussed.

See in major comment my comment about multi pregnancies.

Limitations paragraph need to be more detail, including discussion about potential methodological issues, need to take into account nutritional data and the robustness of the findings.
