# Supplementary material for: Prevalence of low birth weight and its associated factor at birth in Sub-Saharan Africa: A generalized linear mixed model
Source: PLoS One. 2021 Mar 11;16(3):e0248417. doi: 10.1371/journal.pone.0248417 (PMC7951905; doi:10.1371/journal.pone.0248417)
Supplement: Attachment — Submitted filename: MR14.pdf [file pone.0248417.s003.pdf]

USAID
FROM THE AMERICAN PEOPLE

POTENTIAL BIAS AND SELECTIVITY IN ANALYSES OF CHILDREN BORN IN THE PAST FIVE YEARS USING DHS DATA

DHS METHODOLOGICAL REPORTS 14

SEPTEMBER 2014

This publication was produced for review by the United States Agency for International Development. It was prepared by Shea Rutstein of ICF International.

DHS Methodological Reports No. 14

Potential Bias and Selectivity in Analyses of Children Born in the Past Five Years Using DHS Data

Shea O. Rutstein

ICF International

Rockville, Maryland USA

September 2014

Corresponding author: Shea O. Rutstein, International Health and Development, ICF International, 530 Gaither Road, Suite 500, Rockville, MD 20850; Phone: 301-572-0950; Fax: 301-407-6501; Email: Shea.Rutstein@icfi.com

Editor: Bryant Robey
Document Production: Yuan Cheng

This study was carried out with support provided by the United States Agency for International Development (USAID) through the MEASURE DHS project (#GPO-C-00-08-000080-00). The views expressed are those of the author and do not necessarily reflect the views of USAID or the United States Government.

The DHS Program assists countries worldwide in the collection and use of data to monitor and evaluate population, health, and nutrition programs. For additional information about The DHS Program contact DHS Program, ICF International, 530 Gaither Road, Suite 500, Rockville, MD 20850, USA; phone: 301-407-6500, fax: 301-407-6501, email: reports@dhsprogram.com, Internet: www.dhsprogram.com.

Recommended citation:

Rutstein, Shea O. 2014. *Potential Bias and Selectivity in Analyses of Children Born in the Past Five Years Using DHS Data.* DHS Methodological Reports No. 14. Rockville, Maryland, USA: ICF International.

Contents

Contents iii

Tables v

Figures v

Preface vii

Abstract ix

Executive Summary xi

1. Introduction and Purpose 1

2. Characteristics of Most-Recently-Born Children and Non-Most-Recently-Born Children Compared with All Children Born in the Most Recent Five-Year Period 3

3. Biases in the Effects of Subsequent Inter-Pregnancy Intervals on Nutritional Status and Mortality under Age 5 7

3.1 Nutritional Status of Children under Age 5 7

3.2 Under-5 Mortality by Succeeding Birth-to-Conception Interval 10

4. Conclusions and Further Research Needed 13

References 15

Appendix 17

Tables

Table 1. Children born in the five years preceding the survey living at the time of the survey, 45 DHS surveys 5

Table 2. Birth interval by age of child 7

Table 3. Percentage of children last born and with succeeding birth-to-birth intervals, mean succeeding interval length, height-for-age standard deviation, and percentage stunted by child's age, for living children born in the five years preceding the survey, 40 DHS surveys (255,760 children) 9

Table 4. Excluded deaths by length of succeeding interval, children born 1 to 15 years prior to survey, 52 DHS surveys 2006-2012 12

Appendix Table A1. Types of DHS data available on children, by restriction 17

Figures

Figure 1. Mean height-for-age z-score (WHO), by age of child 8

Figure 2. Proportion stunted, by age of child 8

Figure 3. Child mortality by length of succeeding birth interval, children born 1 to 15 years prior to survey, 52 DHS surveys 2006-2012 11

Preface

The Demographic and Health Surveys (DHS) Program is one of the principal sources of international data on fertility, family planning, maternal and child health, nutrition, mortality, environmental health, HIV/AIDS, malaria, and provision of health services.

One of the objectives of The DHS Program is to continually assess and improve the methodology and procedures used to carry out national-level surveys as well as to offer additional tools for analysis. Improvements in methods used will enhance the accuracy and depth of information collected by The DHS Program and relied on by policymakers and program managers in low- and middle-income countries.

While data quality is a main topic of the DHS Methodological Reports series, the reports also examine issues of sampling, questionnaire comparability, survey procedures, and methodological approaches. The topics explored in this series are selected by The DHS Program in consultation with the U.S. Agency for International Development.

It is hoped that the DHS Methodological Reports will be useful to researchers, policymakers, and survey specialists, particularly those engaged in work in low- and middle-income countries, and will be used to enhance the quality and analysis of survey data.

Sunita Kishor

Director, The DHS Program

Abstract

This paper investigates potential biases in analysis of data for only last-born children or non-last-born children instead of all children, and in the analysis of succeeding birth or pregnancy intervals, in both the five-year period preceding the Demographic and Health Surveys (DHS) surveys and for longer periods of time. Beyond the usual considerations of omission of births and transference of births across questionnaire age boundaries, using data for only last-born children or non-last-born children instead of for all children born in the five years preceding the survey can result in biased research findings. Unfortunately, for certain child health outcomes, some DHS data are only collected for last-born children, and other data only for non-last-born. The correction of one bias may create another bias, as in the estimation of mortality risks by succeeding interval. Given the likely negative effects on the health and well-being of children whose birth is followed quickly by another birth, substantial efforts should be made to try to overcome these biases.

Executive Summary

Many studies have shown the deleterious relationships between the length of preceding inter-birth or inter-pregnancy intervals and the pregnancy, birth, and child following the interval. The conception and birth of another child after a short period of time would also be expected to negatively affect the earlier-born child. These considerations have led researchers to attempt to use data from The Demographic and Health Surveys (DHS) Program to investigate health outcomes linked to the subsequent birth or pregnancy interval. Some of the outcomes of interest have data collected only for the five years preceding the survey (e.g. current nutritional status and vaccination rates), while others can use earlier data (for example, child mortality). However, the nature of the problem and the structure of DHS data can lead to serious biases in such analyses. It is the purpose of this Methodological Report to describe and illustrate some of these biases.

In 45 recent DHS surveys, of the nearly half a million children born in the five years preceding the survey, over 70 percent are the most recently born (last birth). The demographic and social characteristics of last-born children differ from those of all children born in the five-year year period preceding the survey. Last-born children are slightly less likely than non-last-born children to be of low birth order. Forty-seven percent of last-born children are of birth orders 1 or 2 compared with 51 percent of non-last-born children. Last-born children are more likely to be male than the non-last-born, 51.6 versus 50.0 percent, respectively. Last-born living children tend to be younger than non-last-born children: about 24 percent of last-born children are age 3-4 compared with 70 percent of the non-last-born.

Mother's age at birth is about two years older for last-born children compared with non-last-born children. Surprisingly, preceding birth-to-birth intervals are much longer for last-born children, with a mean 9.5 months greater (for children of birth order 2 or higher). Comparing last-born children with non-last-born children for social characteristics, there are also surprising differences. Non-last-born children are more likely to live in rural areas, to have been born at home, to have mothers with less education, and to come from somewhat poorer households.

The study of the effects of succeeding inter-pregnancy intervals on children's nutritional status requires that the children be born within the five years preceding the survey, and the next child (if any) would need to be conceived between the date of the index child's birth and the date of the interview. For an index child age 54 months at the time of the survey, for example, the maximum subsequent interval would be approximately 43 months; for an index child age 18 months the maximum subsequent interval would be about 7 months. Thus there is a strong relationship between the age of the index child and the possible length of the subsequent interval.

The older the index child, the longer the inter-pregnancy interval can be, with the mean length rising from 10 to 32 months. On the other hand, chronic nutritional status and underweight increase with the age of children, up to about age 24 months. Therefore, younger children have shorter subsequent intervals on average and also are less likely to have chronic undernutrition, creating a strong bias in the analysis of the relationship between subsequent interval length and chronic nutritional status.

The study of under-5 mortality in relation to the length of the succeeding intergenesic interval is fraught with potential bias from a shortened duration of breastfeeding and the desire to replace a child who died. Excluding all deaths of children who die before the next child is conceived can control for these biases. However, this control could introduce another source of bias: the longer the succeeding birth-to-conception interval, the more deaths and deaths at older ages are excluded. The percent of under-5 deaths prior to a succeeding conception that are excluded from analyses increases as the interval lengthens.

Slightly more than half of deaths are excluded when the interval is less than six months, increasing to 83 percent for reference intervals of 36-47 months, and up to 96 percent for intervals of eight or more years.

Beyond the usual considerations of omission of births and transference of births across questionnaire age boundaries, using data for last-born children or non-last-born children instead of data for all children born in the five years preceding the survey can result in biased research findings. Unfortunately, some DHS data are only collected for last-born children, and other data only for non-last-born children. The correction of one bias may create another bias, as in the estimation of mortality risks by succeeding interval.

Further thought and research are needed on how to overcome these biases for analysis of last children and succeeding intervals. Given the likely negative effects on the health and well-being of children whose birth is followed quickly by another, substantial efforts should be made to try to overcome these biases.

1. Introduction and Purpose

Many studies have shown the deleterious relationships between the length of preceding inter-birth or inter-pregnancy intervals and the pregnancy, birth, and child that follows the interval. Outcomes that have been investigated include miscarriage and stillbirth; complications of pregnancy and delivery; low birth weight; neonatal; infant; and child mortality; and poor nutritional status.

In a review of 58 observational studies, Conde–Agudelo and colleagues identified several plausible causal mechanisms, including folic acid deficiency, cervical insufficiency, vertical transmission of infections, inadequate breastfeeding, and sibling competition (Conde–Agudelo, Rosas–Bermúdez, and Kafury–Goeta 2006). For an in–depth discussion of the literature on this relationship, see previous studies by Rutstein (Rutstein 2005; Rutstein 2008). Several studies demonstrated that even after adjusting for a variety of confounding factors, the effect of birth interval on young child mortality persists (Alam 1995; Alam and David 1998; Bhalotra and van Soest 2006; Conde–Agudelo, Rosas–Bermúdez,and Kafury–Goeta 2006; DaVanzo et al. 2008; Hosseinpoor et al. 2006; Koenig et al. 1990; Miller et al.1992; Mozumder et al. 1998; Zenger 1993).

In addition to the effects of preceding birth interval on child survival, studies have documented a strong relationship between the length of the preceding birth interval and chronic and general undernutrition such that nutrition outcomes are poorer for children with shorter preceding birth intervals (Dewey and Cohen 2007; Rutstein 2005). A recent study by Rutstein and Winter (2014) demonstrated the deleterious effects of combinations of preceding intergenesic intervals, birth order, and mother's age at birth on childhood mortality and nutritional status.

The conception and birth of another child after a short period of time would also seem to negatively affect the earlier-born child at the start of the intergenesic interval. The earlier child's breastfeeding could be cut short, the mother may not have a chance to recover from the earlier pregnancy, birth, and breastfeeding, and care and attention given to the earlier child may be reduced. These considerations have led researchers to attempt to use data from the Demographic and Health Surveys (DHS) to investigate health outcomes linked to the subsequent birth or pregnancy interval. However, the nature of the problem and the structure of DHS data can lead to serious biases in these studies. It is the purpose of this paper to describe and illustrate some of these biases, which are hard to overcome in these kinds of data sets. Appendix Table A1 describes the types of data that are available for children and their restriction by age (or time since birth), birth order, survival status, and other data type.

First, the use of information pertaining to the most-recently-born (last-born) child in the five years preceding the survey is compared with that of all children born in the most recent five-year period, and with children born in the fifteen-year period preceding the survey. Also, children born in the five years preceding the survey with a younger sibling or with the conception of a younger sibling (i.e., those with a succeeding interval) are compared with all children born in the most recent five-year period. Second, factors affecting the nutritional status of children under age 5 that are related to succeeding intervals are studied to evaluate their possibilities of causing bias in studies relating succeeding intervals to the nutritional status of children under age 5. Third, biases that may be present in the relationship between mortality of children under age 5 and succeeding intervals are examined.

2. Characteristics of Most-Recently-Born Children and Non-Most-Recently-Born Children Compared with All Children Born in the Most Recent Five-Year Period

The demographic and social characteristics of most-recently-born children differ from those of all children born in the five-year year period preceding the survey. Studied here are birth order, sex, age at time of survey, mother's age at birth, length of the preceding birth interval, survival of the preceding child, area of residence, delivery in a health facility, mother's level of education, and household wealth.

Table 1 presents selected demographic and social characteristics of children born in the five years preceding the survey and alive at the time of the survey by whether the child is the most recently born. Of the near half a million children born in the preceding five years in 45 recent DHS surveys,[1] over 70 percent are the most recently born (the last birth). These children differ somewhat in many ways from under-5 children who are not the last birth. However, due to the large representation of last-born children among all under-5 children, differences between the last-born and all children are lower. Therefore, the discussion will focus on the differences between last-born and non-last-born children.

Last-born children are slightly less likely than non-last-born children to be of low birth order. Forty-seven percent of last-born children are of birth orders 1 or 2 compared with 51 percent of non-last-born children. Mean birth order is 0.2 children higher among the last-born. Last-born children are more likely to be male than the non-last-born, 51.6 versus 50.0 percent, respectively. This result is surprising in that the sex ratio of non-last-born children is 100. Somewhat less surprising is that there are more males among the last-born (sex ratio of 107). This difference may be due to a preference for male children among couples who stop having children. For all children under age 5, the sex ratio is 105, the expected sex ratio at birth.

Last-born living children tend to be younger than non-last-born children. While there is an approximate balance of all children under age 5, at around 20 percent for each single year of age, only about 24 percent of last-born children are age 3-4 compared with 70 percent of non-last-born children. This striking difference may cause substantial bias when analyses select just last-born or non-last-born children instead of all children. The time since birth also shows that non-last-born children were born about 1.7 years earlier than last-born children. The difference between the current age and time since birth distributions is due to the greater percentage of deaths among non-last-born children, at 11 percent versus 4 percent for last-born children.

The age at birth of the mother is somewhat older for last-born children, by about two years, compared with that of the mothers of non-last-born children. Forty-three percent of last-born children were born to mothers under age 25 compared with 55 percent of non-last-born children.

Preceding birth-to-birth intervals are surprisingly much longer for last-born children, with a mean 9.5 months greater (for children of birth order 2 or higher). This difference may also bias results of analyses if

[1] Albania 2008-09, Armenia 2010, Azerbaijan 2006, Bangladesh 2011, Benin 2006, Bolivia 2008, Burkina Faso 2010, Burundi 2010, Cambodia 2010, Cameroon 2011, Colombia 2010, Congo Democratic Republic 2007, Dominican Republic 2007, Egypt 2008, Ethiopia 2011, Ghana 2008, Guyana 2009, India 2005-06, Indonesia 2007, Jordan 2007, Kenya 2008-09, Lesotho 2009, Liberia 2007, Madagascar 2008-09, Malawi 2010, Mali 2006, Mozambique 2011, Namibia 2006-07, Nepal 2011, Niger 2006, Nigeria 2008, Pakistan 2006-7, Peru 2012, Philippines 2008, Rwanda 2010, Sao Tome and Principe 2008-09, Senegal 2010-11, Sierra Leone 2008 ,Swaziland 2006-07, Tanzania 2010, Timor-Leste 2009 ,Uganda 2011, Ukraine 2007, Zambia 2007, and Zimbabwe 2010-11

only one of the two groups of children is taken to represent all children under age 5.[2] However, the percentage of children whose next older sibling died is not much different between the two groups.

Comparing last-born children with non-last-born children for social characteristics, there are surprising differences. Non-last-born children are more likely to live in rural areas, are more likely to have been born at home instead of in a health facility, have mothers with lower levels of education, and come from somewhat poorer households. The differences between last-born children and non-last-born children may be partly due to differences in fertility levels between countries and areas within countries, as in higher fertility areas (such as rural areas) women are more likely to have had more than one child in the five years preceding the survey.

It is therefore seen that selecting just last-born children or just non-last-born children among the children under age 5 may result in several sources of bias if it is assumed that the results would be the same as for all children. Due to the preponderance of last-born children among all children, the biases in using last-born children are less than those in using non-last-born children. However, the type of analysis undertaken and data available may require using one group or the other instead of all children under age 5. For example, DHS now collects many prenatal and postnatal care indicators only for last-born children. Given that far fewer births of non-last-born children are delivered in a health facility, biased conclusions may result. Dietary information is collected for almost no non-last-born children. If these children were weaned early, their nutritional status could be different from those of last-born children.

[2] The lengths of the succeeding birth-to-birth intervals cannot be compared between the groups since last-born children have no such interval.

Table 1. Children born in the five years preceding the survey living at the time of the survey, 45 DHS surveys

	All children	Last-born children	Non-last-born children
**Number**	433,073	308,724	124,349
**Percent**	100.0	71.3	28.7
**Birth order**			
1	26.0	24.4	30.1
2	22.0	22.4	20.8
3	16.0	16.3	15.1
4	11.5	11.7	11.1
5	8.2	8.2	8.2
6	5.9	6.0	5.7
7+	10.4	11.0	9.0
Mean	3.3	3.3	3.1
**Sex of child**			
Male	51.1	51.6	50.0
Female	48.9	48.4	50.0
**Current age of child (years)**			
0	19.8	27.7	0.0
1	18.7	25.2	2.6
2	18.6	19.5	16.1
3	18.8	13.6	31.8
4	18.1	10.0	38.1
Died	6.1	3.9	11.4
**Time since birth (years)**			
0	20.5	28.8	0.0
1	19.8	26.3	3.6
2	19.9	20.3	18.8
3	20.2	14.2	35.3
4	19.5	10.4	42.2
Mean	2.0	1.5	3.2
**Mother's age at birth**			
<18	6.9	6.0	9.3
18-24	39.7	37.3	45.6
25-29	40.8	42.2	37.4
30-39	9.0	10.1	6.1
40+	3.6	4.5	1.5
Mean	26.1	26.6	24.7
**Preceding birth-to-birth interval**			
<24 months	15.3	13.6	19.5
24-35 months	24.6	23.7	26.8
36-47 months	14.7	15.4	13.0
48+ months	19.4	23.0	10.6
First birth-no preceding interval	26.0	24.4	30.1
Mean	40.9	43.5	34.0

(Continued...)

Table 1. – *Continued*

	All children	Last-born children	Non-last-born children
**Preceding child death**			
Percent with a death	5.8	5.5	6.7
Percent without a death or no preceding child	94.2	94.5	93.3
**Succeeding birth-to-birth interval**			
<24 months	10.0	na	10.0
24-35 months	12.7	na	12.7
36-47 months	5.0	na	5.0
48+ months	1.0	na	1.0
Most recent birth-no succeeding interval	71.3	na	71.3
Mean	27.8	na	27.8
**Area of residence**			
Urban	33.2	35.3	28.0
Rural	66.8	64.7	72.0
**Place of child's birth**			
Non-medical	42.5	39.4	50.4
Medical	57.5	60.6	49.6
**Mother's education in years**			
0	35.3	32.6	41.9
1-6	27.9	27.6	29.9
7-12	30.6	32.8	24.9
12+	6.2	7.0	4.3
Mean	5.0	5.3	4.1
**Wealth Index**			
Value			
Mean	-0.2	-0.1	-0.3
Median	-0.4	-0.3	-0.5
Quintiles			
Poorest	24.5	23.6	28.4
Poorer	21.4	20.9	22.7
Middle	20.2	19.8	19.9
Richer	18.5	18.6	16.8
Richest	15.4	17.1	12.2

3. Biases in the Effects of Subsequent Inter-Pregnancy Intervals on Nutritional Status and Mortality under Age 5

3.1 Nutritional Status of Children under Age 5

The study of the effects of succeeding inter-pregnancy intervals looks at children's nutritional status according to the length of time between the index child's birth and the conception of the next live-born child, if any. Given that children studied need to have been born within the five years preceding the survey, the next child (if any) would need to be conceived between the date of the index child's birth and the date of interview. For an index child age 54 months, the maximum subsequent interval would be approximately 43 months; for an index child age 18 months, the maximum subsequent interval would be about 7 months. Thus there is a very strong relationship between the age of the index child and the length of the subsequent interval. Table 2 demonstrates this relationship.

Table 2. Birth interval by age of child

		Succeeding birth interval		
**Current age of child in years**	**N**	**Minimum**	**Maximum**	**Mean**
0	8	9	11	10.00
1	1,932	9	23	16.43
2	12,532	9	35	23.30
3	24,460	8	47	28.34
4	29,155	8	59	31.52

The older the index child, the longer can be the inter-pregnancy interval, with the mean length rising from 10 to 32 months. On the other hand, chronic nutritional status and underweight increase with the age of children up to about age 24 months. In Figure 1, children's height-for-age z-score decreases with increasing age until about age 20 months. In Figure 2 the percentage of children who are stunted increases with the child's age up to about 24 months. Therefore, younger children have shorter subsequent intervals on average and also have less chronic undernutrition, creating a strong bias in the analysis of the relationship between subsequent interval length and chronic nutritional status.

Figure 1. Mean height-for-age z-score (WHO), by age of child

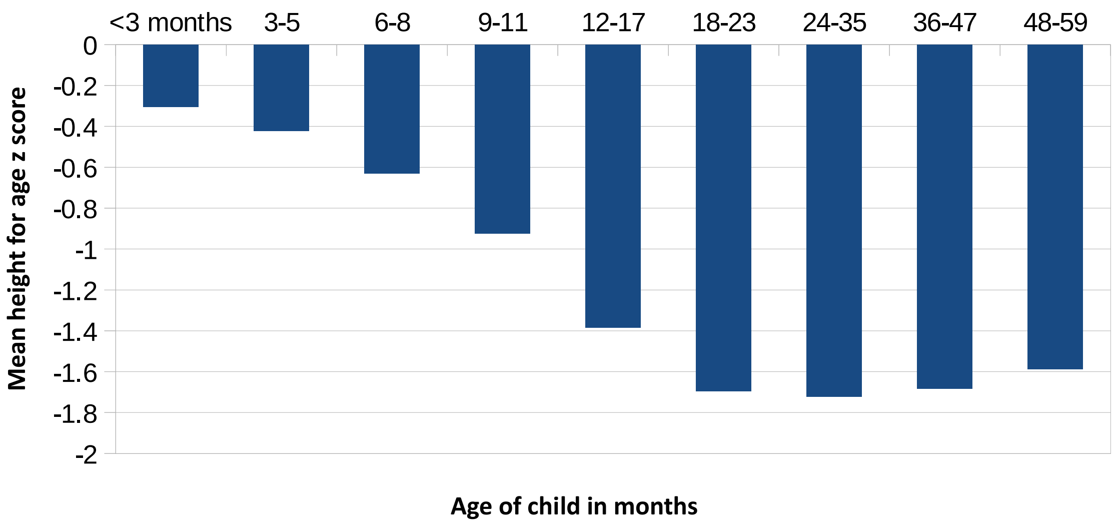

Figure 2. Proportion stunted, by age of child

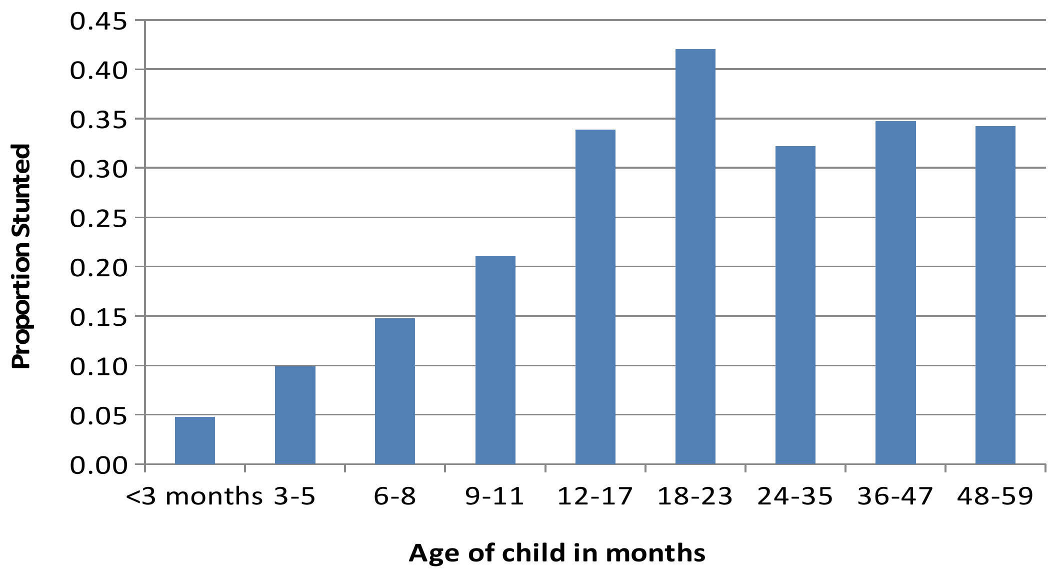

Table 3 below summarizes the confounding that occurs between succeeding interval and chronic undernutrition by age of the index child. Children younger than 18 months have no or almost no succeeding births. For children 18 months or older, the proportion with a succeeding birth increases rapidly, as do the proportions for children with a succeeding birth interval between 12 and 23 months and between 24 and 35 months. However, there are no birth intervals 48 months or longer. The mean interval length also increases rapidly with the child's age. As noted above, the average height-for-age z-score decreases and the percentage stunted increases with the child's age, creating a serious confounder for examining the relationship between succeeding interval and chronic nutritional status.

Table 3. Percentage of children last born and with succeeding birth-to-birth intervals, mean succeeding interval length, height-for-age standard deviation, and percentage stunted by child's age, for living children born in the five years preceding the survey, 40 DHS surveys (255,760 children)

	Child's age in groups									
	<3 months	3-5 months	6-8 months	9-11 months	12-17 months	18-23 months	24-35 months	36-47 months	48-59 months	Total
Child is last-born	100%	100%	100%	100%	99%	94%	75%	52%	40%	73%
Following birth to birth interval less than 12 months	0%	0%	0%	0%	1%	5%	7%	8%	9%	5%
Following birth to birth interval 12 to 23 months	0%	0%	0%	0%	0%	1%	17%	25%	25%	13%
Following birth to birth interval 24 to 35 months	0%	0%	0%	0%	0%	0%	1%	15%	18%	25%
Following birth to birth interval 48 or more months	0%	0%	0%	0%	0%	0%	0%	0%	0%	0%
Succeeding birth interval				10.0	13.3	17.2	23.3	28.3	31.5	28.4
Ht/A standard deviations (according to WHO)	-0.31	-0.42	-0.63	-0.93	-1.39	-1.70	-1.72	-1.68	-1.59	-1.42
Stunted (WHO)	15%	16%	19%	24%	35%	43%	42%	40%	36%	35%

Another serious confounder is breastfeeding. Unless they are postpartum abstinent or using postpartum contraception, mothers who stop breastfeeding are more likely to have a short succeeding interval. The nutritional impact will depend on what age the weaning occurred and what foods the child received after weaning. On the other hand, women who become pregnant while breastfeeding are very likely to stop, and so short succeeding intervals may cause early weaning. Most DHS surveys do not possess data on the duration of breastfeeding of the index child with a succeeding interval, and the early DHS surveys that asked about duration of breastfeeding of non-last-born children had data that were severely heaped, making them useless for analysis.

3.2 Under-5 Mortality by Succeeding Birth-to-Conception Interval

The study of under-5 mortality in relation to the length of the succeeding intergenesic interval is fraught with potential for bias. One such bias relates to breastfeeding. Children who stop breastfeeding early in life face additional mortality risks due to malnutrition and increased prevalence of disease, including diarrhea and acute respiratory infection, especially in low-income settings where milk substitutes are inadequately given and quality health services may not be frequently used. Shortened durations of breastfeeding lead to the early return of postpartum fecundity and are a cause of a short time until the next pregnancy where adequate postpartum contraception and/or abstinence are not practiced. Children who die at young ages also lead to shorter periods of postpartum lactational amenorrhea and thus short succeeding intervals. Another potentially confounding factor is the desire to replace the child who died. Families may want to have another child as soon as possible when a young child dies and thus may not use contraception or prolong periods of postpartum abstinence.

One way to control for these breastfeeding and replacement-desire effects is to exclude all deaths of children who die before the next child is conceived. A child who dies before the next is conceived cannot have been affected by that conception. However, this control could be introducing an additional source of bias: the longer the succeeding birth-to-conception interval, the more deaths and deaths at older ages are excluded. Given that the risk of mortality declines as age at exposure increases, there is a double lowering of mortality of longer intervals compared with shorter intervals. This phenomenon may be the cause of the following results.

Figure 3 shows the adjusted risk of child death by birth-to-conception interval relative to that of the reference interval (36-47 months), using pooled data from 52 DHS surveys for the 15-year period preceding the survey. The relative risk ratio has been adjusted by including a host of characteristics in a Cox hazard regression.[3] Due to the large selection bias that underestimates children's deaths in the reference interval and longer intervals, the relative risks of dying for children with shorter intervals are too high when compared with those of the reference period, giving a misleading impression of the mortality effects of short succeeding intervals.

[3] Controls include sex of child, multiplicity of birth, birth order, mother's age at birth, mother's level of education, type of area of residence, level of household wealth, source of water, type of toilet, and possession of a refrigerator.

Figure 3. Child mortality by length of succeeding birth interval, children born 1 to 15 years prior to survey, 52 DHS surveys 2006-2012

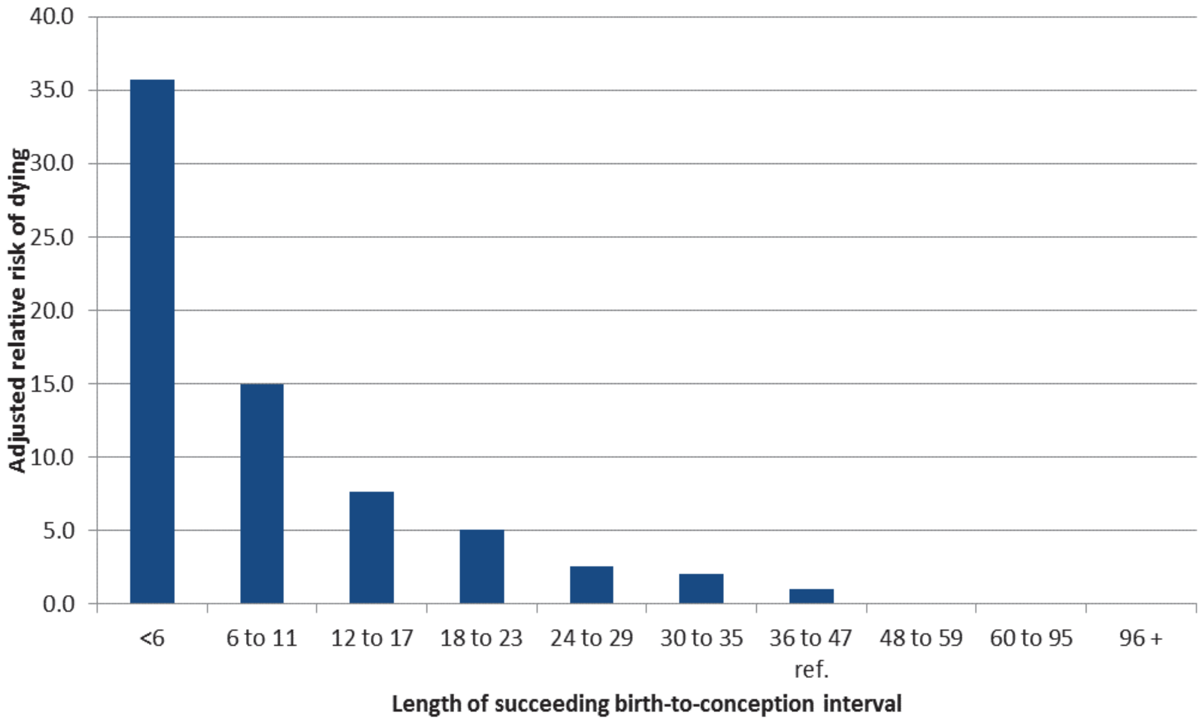

Table 4 shows the percentage of under-five deaths prior to conception of the succeeding child that are excluded from analyses, by succeeding interval for children born in the 15-year period preceding the survey. When the interval is less than six months, slightly more than half of under-5 deaths that occurred prior to conception of the succeeding child are excluded (column 4). These deaths would be mainly neonatal deaths. As the interval lengthens, the percent of under-5 deaths that are excluded increases, reaching 83 percent for the 36-47 month reference interval, and up to 96 percent for intervals of eight years (96 months) or more.

Another way of viewing this bias is the ratio of deaths by interval length to that of the reference category. For an interval of less than 6 months between birth and the succeeding conception, the proportion of deaths included is almost three times higher compared with the reference category, and twice as high for intervals of 12 to 17 months (column 5).

Table 4. Excluded deaths by length of succeeding interval, children born 1 to 15 years prior to survey, 52 DHS surveys 2006-2012

Harmonized succeeding birth-to- succeeding-conception interval (months)	Death before succeeding conception			Percent of deaths excluded	Ratio of percent of deaths included
	Death did not occur before succeeding conception	Death occurred before succeeding conception	Total		
<6	8,334	9,000	17,334	51.9	2.9
6 to 11	6,535	10,583	17,118	61.8	2.3
12 to 17	6,792	13,665	20,457	66.8	2.0
18 to 23	4,029	8,756	12,785	68.5	1.9
24 to 29	1,861	6,168	8,029	76.8	1.4
30 to 35	1,053	3,776	4,829	78.2	1.3
**36 to 47 (ref.)**	**892**	**4,405**	**5,297**	**83.2**	**1.0**
48 to 59	279	2,243	2,522	88.9	0.7
60 to 95	169	2,061	2,230	92.4	0.5
96+	15	354	369	95.9	0.2
Last births	21,287		21,287	0.0	5.9
Total	51,246	61,011	112,257	54.3	2.7

4. Conclusions and Further Research Needed

When researchers use the DHS surveys for the analysis of children born in the five years preceding the survey, they must be very careful to consider and avoid inherent biases that derive from both the data structure and the nature of the analysis. Beyond the usual considerations of omission of births and transference of births across questionnaire age boundaries, particularly the health section and calendar age/date boundaries, using data for last-born children or non-last-born children instead of all children born in the five years preceding the survey will probably result in biased research findings. Unfortunately, some DHS data are only collected for last-born children, for example, children's dietary intake and several child health questions. Other types of research call for using non-last-born children, such as the analysis of current nutritional status by succeeding birth or pregnancy interval, because survey-measured nutritional status of children under age 5 is only relevant for those born in the last five years who have a succeeding interval—that is, non-last-born children under age 5. Also presented is the case that the necessary correction of one bias may incur the creation of another type of bias, as in the estimation of mortality risks by succeeding interval.

Further thought and research are needed on how to overcome these biases for analysis of last-born children and succeeding intervals. Possible solutions are to obtain information on all children instead of just the last-born, collect past nutritional status information, as the Peru Continuous DHS survey does from health cards, and collect follow-up information using cohort panels. Other analytical techniques may also be helpful such as differential weighting, instrumental variable and two-stage regressions,[4] and time-varying covariation for death and next conception. Given the possibly great effects on the health and well-being of children whose birth is followed quickly by that of another, substantial efforts should be made in trying to overcome these biases.

[4] As an example, an analysis using two-stage regression could estimate the level of stunting according to the child's age. Then the predicted level of stunting could be subtracted from the actual level for each child and the difference could be regressed on the succeeding birth-to-pregnancy interval. Control variables (education, wealth, etc.) could be introduced in either the first or the second regression.

References

Alam, N. 1995. “Birth Spacing and Infant and Early Childhood Mortality in a High Fertility Area of Bangladesh: Age–Dependent and Interactive Effects.” *Journal of Biosocial Science* 27(4): 393–404.

Alam, N., and P.H. David. 1998. “Infant and Child Mortality in Bangladesh: Age–Specific Effects of Previous Child’s Death.” *Journal of Biosocial Science* 30(03): 333–48.

Bhalotra, S., and A.V. Soest. 2006. “Birth Spacing and Neonatal Mortality in India: Dynamics, Frailty and Fecundity.” Paper read at Population Association of America, at Los Angeles, California.

Conde–Agudelo, A., A. Rosas–Bermúdez, and A.C. Kafury–Goeta. 2006. “Birth Spacing and Risk of Adverse Perinatal Outcomes: A Meta–Analysis.” *JAMA* 295(15): 1809–23.

DaVanzo, J., L. Hale, A. Razzaque, and M. Rahman. 2008. “The Effects of Pregnancy Spacing on Infant and Child Mortality in Matlab, Bangladesh: How They Vary by the Type of Pregnancy Outcome that Began the Interval.” *Population Studies (Cambridge)* 62(2): 131–54.

Dewey, K.G., and R.J. Cohen. 2007. “Does Birth Spacing Affect Maternal or Child Nutritional Status? A Systematic Literature Review.” *Maternal & Child Nutrition* 3(3): 151–73.

Hosseinpoor, A.R., E. van Doorslaer, N. Speybroeck, M. Naghavi, K. Mohammad, R. Majdzadeh, B. Delavar, H. Jamshidi, and J. Vega. 2006. “Decomposing Socioeconomic Inequality in Infant Mortality in Iran.” *International Journal of Epidemiology* 35(5): 1211–9.

Koenig, M.A., J.F. Phillips, O.M. Campbell, and S. D’Souza. 1990. “Birth Intervals and Childhood Mortality in Rural Bangladesh.” *Demography* 27(2): 251–65.

Miller, J.E., J. Trussell, A.R. Pebley, and B. Vaughan. 1992. “Birth Spacing and Child Mortality in Bangladesh and the Philippines.” *Demography* 29(2): 305–18.

Mozumder, A.K.A., T.T.K. Barkat–e–Khuda, T.T. Kane, and M.S.I. Khan. 1998. *Determinants of Infant and Child Mortality in Rural Bangladesh*. Bangladesh: International Centre for Diarrhoeal Disease Research.

Rutstein, S.O. 2005. “Effects of Preceding Birth Intervals on Neonatal, Infant and Under–Five Years Mortality and Nutritional Status in Developing Countries: Evidence from the Demographic and Health Surveys.” *International Journal of Gynecology & Obstetrics* 89: S7–24.

Rutstein, S.O. 2008. *Further Evidence of the Effects of Preceding Birth Intervals on Neonatal, Infant, and Under–Five–Years Mortality and Nutritional Status in Developing Countries: Evidence from the Demographic and Health Surveys.* DHS Working Papers No. 41. Calverton, Maryland, USA: Demographic and Health Research Division, Macro International.

Rutstein, S.O, and R. Winter. 2014. *The Effects of Fertility Behavior on Child Survival and Child Nutritional Status: Evidence from the Demographic and Health Surveys, 2006 to 2012*. DHS Analytical Studies No. 37. Rockville, Maryland, USA: ICF International.

Zenger, E. 1993. “Siblings’ Neonatal Mortality Risks and Birth Spacing in Bangladesh.” *Demography* 30(3): 477–88.

Appendix

Appendix Table A1. Types of DHS data available on children, by restriction

Questionnaire source of information	Type of data	Restrictions on data		
		Time since birth	Survival	Other
Household schedule	Age, sex, relation to head	All	Living children	
	Marital status	15+ years	Living children	
	Survival and residence of parents	0 to 17 years	Living children	
	Education attainment	5+ years	Living children	
	Education attendance	5 to 24 years	Living children	
	Birth registration	0 to 4 years	Living children	
Household biomarker form	Height, weight	0 to 5 years	Living children	
	Hemoglobin level	6 months to 5 years	Living children	
Women's questionnaire				
Birth history	Date of birth, sex, multiplicity of birth, birth order, survival	All	All	
	Preceding and succeeding birth interval, age of mother at birth	All	All	
	Age at death	All	Dead children	
Maternity section	Wantedness, size and weight at birth, delivery assistance	0 to 4 years	All	All
	Place of delivery, caesarian delivery	0 to 4 years	All	All
	Antenatal care	0 to 4 years	All	Last birth only
	Time in delivery facility, postnatal check of mother, baby	0 to 4 years	All	Last birth only
	Postpartum amenorrhea and abstinence	0 to 4 years	All	All
	Ever breastfed, use of bottle with nipple	0 to 4 years	All	All
	Time to first breastfeed and liquids in first 3 days	0 to 4 years	All	Last birth only
	Still breastfeeding	0 to 4 years	Living children	Last birth only
Child health section	Vaccinations	0 to 4 years	Living children	All
	Illness and treatment	0 to 4 years	Living children	All
	Stool disposal	0 to 4 years	Living children	Youngest living with mother
	Foods and liquids in last 24 hours	0 to 2 years	Living children	Youngest living with mother
Reproductive calendar	Duration of pregnancy, prior termination	0 to 4 years	All	
	Birth as a result of contraceptive failure	0 to 4 years	All	
