# Supplementary material for: Prevalence of low birth weight and its associated factor at birth in Sub-Saharan Africa: A generalized linear mixed model
Source: PLoS One. 2021 Mar 11;16(3):e0248417. doi: 10.1371/journal.pone.0248417 (PMC7951905; doi:10.1371/journal.pone.0248417)
Supplement: Attachment — Submitted filename: PLOS ONE.docx [file pone.0248417.s001.docx]

PLOS ONE

The authors present an important analysis assessing risk factors for low birthweight in Sub-Saharan Africa. The analysis pools recent (2008-2018) DHS surveys from 35 different to represent the sub-continent and specific regions, Central Africa, East Africa, West, Africa, South Africa. The authors find risk factors and protective factors for low birth weight similar to those established in the literature. While there may be some advantage to assessing risk factors by region as the authors aim, there are some concerns with the overall methods used in the manuscript. Further the manuscript would benefit from English language editing. I have provided some suggestions below.

**Major:**

**Methods**

1) A pooled risk factor analysis may be inappropriate with these data. Pooling multiple surveys from 35 countries over a 10-year period results in a poorly defined population. An analysis such as LBW is likely to be overpowered with such a large dataset and so may detect statistical differences that are not clinically meaningful. For example, all of the included risk factors had a pvalue<0.2 and were included in the multivariable analysis. For low birthweight, you likely to have enough power in each individual survey and so don’t benefit from this type of pooling. It would be appropriate however to use weighted data from these studies to estimate relative burden of LBW and the risk factors in the larger region or sub-regions. The authors could consider focusing on just one region and stratifying by country and assess for differences by country.
2) It would be helpful for the authors to provide a flow chart of the data. It would be helpful to know what proportion of respondents were excluded based on missing birthweight and know if the risk factors were similar to those who were included in the study. Please include number of live births, and what proportion had LBW measured.
3) Line 125: Please provide more details about the meta analysis shown in the forest plot. I assume this was fixed effects meta analysis? The forest plot is only a visualization of the analysis.
4) Please specify the levels of hierarchical model, does the model include cluster, country and region?
5) It is not clear how missing data is handled. Was the multivariable model a complete case analysis?
6) Line 121: It is unclear to me if a re-weighted data set was created as is described here. f that is the case please show the re-weighted sample size and numbers throughout. If not, please show weights applied to each country sample in table 1 and other table throughout. Or if the authors applied sampling weights to each analysis, please specify in the methods.

**Results**:

7) Please check the variable for husband education status. There are more responses than there are participants who are married. There should be missing values for the 31904 women who are not married.
8) Similar to 6: Please check variable for preceding birth interval. How is this defined for a woman’s first birth? If they are in the ≥24 months, this could be the reason for the unexpected results.

**Discussion:**

9) Line 203: A major limitation discussed in the methods above, is that this burden estimate is based on mothers who remembered or had documentation of children’s LBW. This factor should be included here.
10) Overall the discussion could be improved to put the results in context for the reader. Consider restricting the discussion to focus on some key results. The conclusion discusses modifiable risk factors. One approach could be to discuss the modifiable risk factors and the context of interventions for these risk factors. Brining in the discussion of preterm and fetal growth restriction may also bring context to these results.
11) All statements of explanation for these findings should be based on the literature and so need to be sited (line 219, 221, 228, 236).
12) Line 236: LBW associated with multiples is well established and it is an oversight here not to mention that multiples are almost always born early, and so increased risk for LBW for that reason.

**Minor:**

**Introduction:**

1) Line 56: Please define LBW abbreviation – the first sentence could read, Low birth weight (LBW) is one of the….
2) Line 61-63: The last sentence in the first paragraph doesn’t seem relevant to this overall summary, and more relevant that the author are based in Ethiopia. I suggest deleting.
3) Line 71 “well facts” should be reworded. Could say are well established.
4) Line 78 Nutritional shouldn’t be capitalized

**Methods:**

5) Line 90-94: Please include rational for why these countries were selected. The list does not include the WHO African region or UN Africa region.
6) Line 97-99: Description of the different DHS data files isn’t necessary, specifying the KR file was used is sufficient.
7) Line 111: the specific coded values is not necessary to include. This line could just say “LWB was defined as a birth weight <2.5kg, and those ≥2.5kg were considered normal and above normal birthweights.”
8) Line 114: “Based on kinds of literature” needs to be edited. It could be revised to say, potential risk factors for LBW were included based on a review of the literature. (Please include a citation).
9) Line 137: If the P-value is used to base the decision on inclusion into the multivariable model, please include P-values with the bi-variate results. Because of the study is over powered for these detections, consider lowering the threshold of what is included in the multivariable analysis. If there were variables considered that did not meet this threshold, they should also be included in the manuscript.

**Results**:

1) Line 155: The rewording the description of highest low birth weight and lowest birthweight to make it easier to read. Consider saying highest LBW prevalence, or lowest rate of LBW births.
2) Line 159: Details of the model comparison do not need to be included in the main analysis results. They could be included in the appendix.
3) Line 176: Careful saying mothers were less likely to low birthweight. Consider revising to say “were less likely to deliver LBW infants than mothers without formal education:
4) Line 182: Please add definitions of Poor, Middle and Rich, and consider using the wealth quintile for comparison. Line 182 should read “among mother’s in the middle and rich wealth categories.”
5) Line 184: type on the confidence interval. Should be 0.91 instead of 0.81 (according to the table)
6) Line 185: Consider rephrasing to say “Mothers who have do not participate making their own”
7) Line 186: Typo – no should be removed: consider rephrasing to say “than mothers who participate in making their own health care decisions” or “than mothers who make their own health care decisions” if that is an appropriate interpretation of the question asked.
8) Line 193: Typo Bing should be being.
9) Line 194: Single should be singleton
10) Table 2: please define media exposure.
11) Table 4: Please add row percent to the overall numbers in each group.
12) Table 4 CORs are missing on the last page for parity, sex of child, type of births, iron supplementation.

**Discussion**

13) Line 206: The statement about Brazil seems irrelevant for a paper focused on SSA- consider removing
14) Line 255: consider the term multiples here instead of multiple pregnancies.

**Considerations**

1) It would be helpful to re-orient the risk factors so they are all risk factors or protective factors to help readability.
2) Consider looking for trends in birthweight by year, may want to control for the year of the DHS survey.
3) Consider combining Table 2 and 4.
