# Supplementary material for: Prevalence of low birth weight and its associated factor at birth in Sub-Saharan Africa: A generalized linear mixed model
Source: PLoS One. 2021 Mar 11;16(3):e0248417. doi: 10.1371/journal.pone.0248417 (PMC7951905; doi:10.1371/journal.pone.0248417)
Supplement: Attachment — Submitted filename: Point by point response.docx [file pone.0248417.s004.docx]

**PLOS ONE**

**Point by point response for editors/reviewers comments**

The manuscript title “**Prevalence of low birth weight and its associated factor at birth in Sub-Saharan Africa: A Generalized Linear Mixed	Model”**

**Manuscript ID:** **PONE-D-20-27726**

**Dear editor/reviewer.**

Dear all,

I would like to thank you for these fair, constructive, building, and improvable comments on this manuscript that would improve the substance and content of the manuscript. We considered each comment and clarification questions of reviewers on the manuscript thoroughly. My point-by-point responses for each comment and question are described in detail on the following pages. Further, the details of changes were shown by track changes in the supplementary document attached.

**Response to Reviewers comments**

**Reviewer #1**

1. The timing of the information is seen in Table 1 as noted in the ‘Data Source’ information. Also assurances should be given of the homogeneity of the sample across the countries

**Author response:-** Thank you very much for your fair and contractive comments. We accept your comment and corrected it accordingly. We included your concern in the revised manuscript which describe the homogeneity of sampling procedure across sub-Saharan Africa countries line 94-95(see the revised manuscript)

**Reviewer #2**

1. There may be some advantage to assessing risk factors by region as the authors aim,

**Author’s response:-** Thank you very much for your concern. There are different research articles done on assessing risk factors by regions(Eastern Africa, Southern Africa, Western Africa, and Central Africa). Our primary objective was assessing factors that represent and can be generalizable at sub-Saharan Africa which consists of the entire four WHO sub-Saharan Africa region(Eastern Africa, Southern Africa, Western Africa, and Central Africa). The 35 sub-Saharan Africa country datasets were appended together for the prediction of low birth weight babies in SSA. The DHS data is a nationally representative survey that collects data on basic health indicators like mortality, morbidity, family planning service utilization, fertility, maternal and child health. The data were derived from the measure DHS program. The DHS has different datasets (men, women, children, birth, and household datasets). For this study, we used the children dataset(KR dataset.)

2. There are some concerns with the overall methods used in the manuscript.

**Authors response:-** Thank you very much for your concern. We would be very happy if the comment was clear and indicate the specific methodological problem. The given comment is crude and blind. We would be very happy if you tell us the methodological problems you observed. The method session is adopted from the procedure of DHS program from across all sub-Saharan Africa countries. We try to use the advanced statistical technique that considers the hierarchical nature of the DHS data i.e Generalized Linear Mixed Effect Model(GLMM). We are open to correct the methodological problems you observed if you indicate us clearly for the improvement of the manuscript.

3. The manuscript would benefit from English language editing. I have provided some suggestions below. See the attachment for more details

**Authors response:-** Thank you very much for your contractive and fair comments for the improvement of the manuscript. We accept your comment and corrected it accordingly. In addition, we gave our manuscript to an English editor and we corrected grammatical and punctuation errors accordingly (see the revised manuscript).

**Reviewer #3**

1. The principal issue of this work is the use of DHS data.

DHS is known as a formidable source of data and give a general overview of sanitary situation of each country (trend of key indicators). However, there are many methodological concerns about accuracy of data collection, population sample and high risk of potential biases: Selection biases (due to different household sampling techniques), information or classification biases (due to self-report data or declared data, medical records, standardization of data collection …). In addition, the methodology can differ between countries, especially concerning the under-five children data. Indeed, two main different methods can be used to collect these data: Most-Recently-Born Children and Non-Most-Recently-Born Children.

I invite the authors to read this critical analysis of the use of DHS data for children (pdf attached), whose conclusion I quote: “When researchers use the DHS surveys for the analysis of children born in the five years preceding the survey, they must be very careful to consider and avoid inherent biases that derive from both the data structure and the nature of the analysis. Beyond the usual considerations of omission of births and transference of births across questionnaire age boundaries, particularly the health section and calendar age/date boundaries, using data for last-born children or non-last-born children instead of all children born in the five years preceding the survey will probably result in biased research findings.

**Authors response:-** Thank you very much for the concerned reviewer. For this study, we used **KR** dataset. This dataset records children with the most recent one rather than all children in the household. We read a pdf you attached us. We included this issue as a limitation after we read the pdf. For this study, we used the most recent birth within five years preceding the survey for each country. Therefore, not to miss leading the reader we included it in the limitation session(see the revised manuscript).

Analysis (statistical issue)

2. Multiple pregnancies (such as twin pregnancies) were included in the same models as a single pregnancy. Multiple pregnancies (MP) are known as a mediator factor of LBW (MP is on the pathway of LBW). Therefore, this can lead to a sur-adjustment. I will be more useful to perform the main analysis after excluding MP and to perform a sensitivity analysis with MP.

**Authors response**:- We conducted the sensitivity analysis according to your recommendation(with and without the multiple pregnancies the result presented as below).

**The sensitivity analysis without MP(multiple pregnancies)**

**Sensitivity analysis with MP (multiple pregnancies ) included**

| Model comparison | With MP(multiple pregnancies) | Without multiple pregnancies | Decision |
| --- | --- | --- | --- |
| LL | -45356 | -46696 | Largest bette |
| Deviance | 90717 | 93392 | Smallest better |

Thank you very much for your concern. We conducted a sensitivity analysis with and without including multiple pregnancies in the model. The Area under the curve (ROC) curves with multiple pregnancies in the model was 62.19% and without Multiple pregnancies in the model was 58.18%. Therefore, including MP in the model is better than excluding it. The likelihood (Largest better)and Deviance(smallest better) also support model with MP included was better than the model without MP. The works of literature also support that multiple pregnancies are a predictor of low birth weight.

3. The use of “Determinants” in the title by the authors is misleading to me. The notion of determinant implies a causal role while the notion of the risk factor or associated factor is broader and refers to a higher probability of disease in exposed subjects. In this work, it is rather “associated factors” that have been highlighted.

**Authors response:-** Thank you very much for your comment. We accept your comment and corrected it accordingly. We change the title to “Prevalence of low birth weight and its associated factor at birth in Sub-Saharan Africa: A Generalized Linear Mixed Model”(see the revised manuscript).

Abstract

4. Lines 47-48: In conclusion of abstract, the authors declared “this study… with significant variations among countries” but any variations or range have been presented in the results paragraph of abstract. Please, correct it.

**Authors response:-** Thank you very much for your contractive comment. We accept your comment and corrected it accordingly line number 48-49 (see the revised manuscript).

Introduction

5. Lines 71-72: 25-30 times seems very high today (substantial progress has been made, even in countries with limited resources). I suggest that the authors further clarify this information, either by adding that this very high risk is for very low birth weight infants, or that the information is several years old.

**Author’s response:-** Thank you very much for your contractive comment. We accept your comment and corrected it accordingly line number 71 (see the revised manuscript).

6. Lines 80-82: There are many studies such as systematic reviews, multicenter pooled analyses (some of which have even been cited by the authors). The rationale for this study therefore needs to be better specified. For the moment, we do not really see the added value or the new knowledge brought.

**Author’s response**:- Thank you very much for your comment. We accept your comment and corrected it accordingly line number 80-87 (see the revised manuscript)

7. Lines 85-86: I am not convinced.

**Authors response:-** Thank you very much for your comment. The intention for this point was the finding of this study is better than systematic review and meta-analysis. The reason was DHS program used the same study design(cross-sectional nature), the same manual for data collection, the variable coding, the same sampling procedure etc… As we know in the systematic review and meta-analysis there may be different study design, data collection tool, different coding of variables, publication bias, etc…..because of this difference we believe that this study finding is better than a systematic review and meta-analysis. We remove this idea from the revised manuscript if it misses leads readers and we substitute other word line number 90-92(see the revised manuscript).

Methods

8. See my major comment about the use of DHS data. I suggest that the authors briefly present the methods used to collect data on children in the DHS to give us an idea of the robustness or otherwise of the data and potential biases.

**Authors response:-** Thank you very much for your concern. For this study, we used KR(kids record dataset). This dataset records the last or the recent child in the family. We read your materials attached us with the comment regarding data analysis, especially for under-five children data. We agree with your concern. There may be a bias result if we did not include all children’s in the family. According to our scenario, we used the most recent child five years preceding the survey for each 35 sub-Saharan Africa countries. So we boldly mentioned this point in the limitation session line (see the revised manuscript)

9. What about children who were not included in this analysis. Do the authors check any potential selection biases?

**Authors response:-** Thank you very much for your constructive comment. The data used for this study was secondary data obtained from DHS program after project proposal was submitted to the DHS program office and permission was obtained from data acrchivest. The dataset records for those children born from women within five years preceding the survey and the most recent one. Within one family there may be more than one child but the KR dataset recorded the most recent one. According to the above scenario, there is no selection bias for this study because the most recent bias was obtained.

10. It will be useful to have a country included in each region.

**Authors response:-** Thank you very much for your contractive comment. We accept your comment and corrected it accordingly see **Table 1** (see the revised manuscript ).

11. How infant weight were measured at birth? Standardization? Declared (or self-report data?) Medical record?

**Authors response:-** Thank you very much for your comment. The birth size was measured using self-report from mother recall, medical records from a nearby health facility, and weights measured at birth. In SSA a small number of children were recorded their weight birth size. Most infant weights were obtained from their mother's self-report. Because of this, we include this point as a limitation because this may result recall bias.

12. Variable definition paragraph is missing in this work, particularly for the clarification of the definition of variables such as “household wealth index”, “women health care decision making autonomy”.

**Authors response:-** Thank you very much for your contractive comment. We accept your comment and corrected it accordingly line 130-137(see the revised manuscript ).

13. What about other important independent variables such as malaria infection or BMI?

**Authors response:-** Thank you very much for your concern. Yes, you are right these variables are important independent variables but malaria infection was not collected in the DHS dataset and BMI has had a high missing value because of this we excluded it from the analysis.

14. ANC: please, define the acronym the first time you use it.

**Authors response:- Authors response:-** Thank you very much for your contractive comment. We accept your comment and corrected it accordingly(see the revised manuscript ).

15. The parameters of mixed logistic regression need to be more detail. Indeed, the authors used multilevel models to take into account the hierarchical structure of data. However, it is important to specify what represents the 1st level (individual?), the 2nd level (regions or countries?). Please, clarify.

**Authors response:-** Thank you very much for your comment. **There are two types of variable in the analysis. Level one varianles include (**maternal education, husband education, maternal age, mother marital status, household wealth index, maternal occupation status, women health care decision making autonomy, media exposure, number of ANC visit, preceding birth interval, parity, sex of the child, type of birth and iron supplementation**)** and level two variables (residence , region and country)

Results

16. Table 2: West Africa weighted frequency is 12170, but Benin (which is West African country) only provide 13909 participants. Please, clarify.

**Authors response:-** Thank you very much for your comment. We corrected it accordingly(see the revised manuscript).

17. Table 4: It will be useful to have also percentage in normal and LBW columns.

**Authors response:-** Thank you very much for your comment. We corrected it accordingly(see the revised manuscript)

18. Lines 162-163: “Women from a cluster … lower birth weight”. This sentence is a bit confusing. Please, clarify.

**Authors response:-** Thank you very much for your comment. We accept your comment and corrected it accordingly(see the revised manuscript)

Discussion

19. Line 197: “8.9% to 10.3%”, please include this result in results session also.

**Authors response:-** Thank you very much for your comment. We corrected it accordingly(see the revised manuscript)

20. In general, the discussion section need to be more focus on comparison of this study with other multicentric pooled analysis and meta-analysis studies in sub-Saharan Africa, instead of comparison of single center or no-pooled analysis studies.

**Authors response:-**Thank you very much for your comment. We accept your comment and corrected it accordingly(see the revised manuscript).

21. Lines 199-207: For me, these comparisons are not relevant (single center studies vs. pooled multicenter study).

**Authors response:-** Thank you very much for your comment. We accept your comment and corrected it accordingly(see the revised manuscript).

22. Lines 214-224: All this paragraph is a speculation. What about nutritional factors in this study. Iron supplementation was not associated with LBW in this study. Why? A discussion of this negative finding will be useful. Why didn't the authors consider other indicators of nutritional status such as BMI? If nutritional status plays an important role in the occurrence of LBW, all of the above should be discussed.

**Authors response:-** Thank you very much for your comment. We accept your comment and corrected it accordingly(see the revised manuscript).

23. See in major comment my comment about multi pregnancies.

**Authors response**:- Thank you very much for your comment. We try to address this issue in number two of your question.

24. Limitations paragraph need to be more detailed, including discussion about potential methodological issues, need to take into account nutritional data and the robustness of the findings.

**Author response:-** Thank you very much for your comment. We revised the limitation section of this manuscript after your suggestion including missed variables in the dataset and methodological issues that readers should take into account(see the revised manuscript).
