# Supplementary material for: Prevalence of low birth weight and its associated factor at birth in Sub-Saharan Africa: A generalized linear mixed model
Source: PLoS One. 2021 Mar 11;16(3):e0248417. doi: 10.1371/journal.pone.0248417 (PMC7951905; doi:10.1371/journal.pone.0248417)
Supplement: Attachment — Submitted filename: PLOS ONE v3.docx [file pone.0248417.s005.docx]

PLOS ONE

The authors present an important analysis assessing risk factors for low birthweight in Sub-Saharan Africa. The analysis pools recent (2008-2018) DHS surveys from 35 different to represent the sub-continent and specific regions, Central Africa, East Africa, West, Africa, South Africa. The authors find risk factors and protective factors for low birth weight similar to those established in the literature. While there may be some advantage to assessing risk factors by region as the authors aim, there are some concerns with the overall methods used in the manuscript. Further the manuscript would benefit from further English language editing. It appears the authors did not receive the detailed comments and suggestions below after the first review.

**Major:**

**Methods**

1) A pooled risk factor analysis may be inappropriate with these data. Pooling multiple surveys from 35 countries over a 10-year period results in a poorly defined population. An analysis such as LBW is likely to be overpowered with such a large dataset and so may detect statistical differences that are not clinically meaningful. For example, all of the included risk factors had a pvalue<0.2 and were included in the multivariable analysis. For low birthweight, you likely to have enough power in each individual survey and so don’t benefit from this type of pooling. It would be appropriate however to use weighted data from these studies to estimate relative burden of LBW and the risk factors in the larger region or sub-regions. The authors could consider focusing on just one region and stratifying by country and assess for differences by country.
2) It would be helpful for the authors to provide a flow chart of the data. It would be helpful to know what proportion of respondents were excluded based on missing birthweight and know if the risk factors were similar to those who were included in the study. Please include number of live births, and what proportion had LBW measured.
3) Line 101-102: Please provide DHS or other citations (or an appendix) referencing the sampling procedure in each country is the same. It is very likely that the sampling strategies were different in different countries.
4) Line 125: Please provide more details about the meta analysis shown in the forest plot. I assume this was fixed effects meta analysis? Is this how the pooled prevalence was calculated? Please clarify. The forest plot is only a visualization of the analysis.
5) It seems more levels of the hierarchical model may be needed. Consider clustering on the woman, household, cluster and country. (region might not be necessary).
6) It is not clear how missing data is handled. Was the multivariable model a complete case analysis?

**Results**:

7) Line: 166: the text says 202878 mothers were included, yet the variables in table 2 sub to 203,466. Is this due to the weighting? Other numbers in this paragraph also don’t match the tables.
8) Line 166: I think you mean West Africa and not East Africa?
9) Table 2 shows the same number of women and the same number of children. If all children born to women in the last 5 years are included in the analysis, I would expect there to be more children included in the analysis than women included. Please clarify the discrepancy and add the number of women and number of children included in the analysis separately in the text (the flow chart in suggestion 2 would also help this point).
10) Sample sizes from table 1 don’t match those listed in figure1
11) The variable of husband’s education is misrepresented. 31904 women who are not married should have husband’s education as missing.
12) Please clarify the meaning of “No” for education status. Do you mean less than primary education?
13) Similar to 6: Please check variable for preceding birth interval. How is this defined for a woman’s first birth? If they are in the ≥24 months, this could be the reason for the unexpected results.
14) Table 4: Please present column % instead of row % for comparison between normal and low birthweight.

**Discussion:**

15) Overall the discussion could be improved to put the results in context for the reader. Consider restricting the discussion to focus on some key results. The conclusion discusses modifiable risk factors. One approach could be to discuss the modifiable risk factors and the context of interventions for these risk factors. Brining in the discussion of preterm and fetal growth restriction may also bring context to these results.
16) All statements of explanation for these findings should be based on the literature and so need to be sited (line 227, 233, 237, 240, 249, 252, 254. 258).
17) Line 236: LBW associated with multiples is well established and it is an oversight here not to mention that multiples are almost always born early, and so increased risk for LBW for that reason.

**Minor:**

In response to reviewer 3’s suggestions, remove the word determinants in in line 38, 184

**Introduction:**

1) Line 56: Please define LBW abbreviation – the first sentence could read, Low birth weight (LBW) is one of the….
2) Line 61-63: The last sentence in the first paragraph doesn’t seem relevant to this overall summary that is at the SSA level. I suggest deleting or replacing high and low estimates for the region.
3) Line 71 “well facts” should be reworded. Could say are well established.
4) Line 78 Nutritional shouldn’t be capitalized.
5) Please provide citations for other LBW studies, meta analyses systematic reviews that referenced in line 83.
6) 89-92. I am not convinced region level estimates are very informative for interventions and policy decisions.

**Methods:**

7) Line 96-101: Please include rational for why these countries were selected. The list does not include the WHO African region or UN Africa region.
8) Line 111: the specific coded values is not necessary to include. This line could just say “LWB was defined as a birth weight <2.5kg, and those ≥2.5kg were considered normal and above normal birthweights.” – this suggestion could be applied throughout the manuscript.
9) Line 122: “Based on kinds of literature” needs to be edited. It could be revised to say, potential risk factors for LBW were included based on a review of the literature. (Please include a citation).
10) Line 156: If the P-value is used to base the decision on inclusion into the multivariable model, please include P-values with the bi-variate results. Because of the study is over powered for these detections, consider lowering the threshold of what is included in the multivariable analysis. If there were variables considered that did not meet this threshold, they should also be included in the manuscript.

**Results**:

1) Line 172: Please reword the description of highest low birth weight and lowest birthweight to make it easier to read. Consider saying highest LBW prevalence, or lowest rate of LBW births.
2) Line 193: Careful saying mothers were less likely to low birthweight. Consider revising to say “were less likely to deliver LBW infants than mothers without formal education:
3) Line 201: type on the confidence interval. Should be 0.91 instead of 0.81 (according to the table)
4) Line 210: Typo Bing should be being.
5) Table 2: please define media exposure.
6) Table 4 CORs are missing on the last page for parity, sex of child, type of births, iron supplementation.

**Discussion**

7) Line 229: The statement about Brazil seems irrelevant for a paper focused on SSA- consider removing. The authors could consider comparing to other regions instead.
8) consider the term multiples throughout instead of multiple pregnancies.

**Considerations**

1) It would be helpful to re-orient the risk factors so they are all risk factors or protective factors to help readability.
2) Consider looking for trends in birthweight by year, may want to control for the year of the DHS survey.
3) Consider combining Table 2 and 4.
