# Supplementary material for: Prevalence of low birth weight and its associated factor at birth in Sub-Saharan Africa: A generalized linear mixed model
Source: PLoS One. 2021 Mar 11;16(3):e0248417. doi: 10.1371/journal.pone.0248417 (PMC7951905; doi:10.1371/journal.pone.0248417)
Supplement: Attachment — Submitted filename: Point by point response.docx [file pone.0248417.s006.docx]

**PLOS ONE**

**Point by point response for editors/reviewers comments**

The manuscript title “**Prevalence of low birth weight and its associated factor at birth in Sub-Saharan Africa: A Generalized Linear Mixed	Model”**

**Manuscript ID:** **PONE-D-20-27726**R1

**Dear editor/reviewer.**

Dear all,

I would like to thank you for these fair, constructive, building, and improvable comments on this manuscript that would improve the substance and content of the manuscript. We considered each comment and clarification questions of reviewers on the manuscript thoroughly. My point-by-point responses for each comment and question are described in detail on the following pages. Further, the details of changes were shown by track changes in the supplementary document attached.

**Response to Reviewers comments**

**Reviewer #1**

Major:

Methods

1) A pooled risk factor analysis may be inappropriate with these data. Pooling multiple surveys from 35 countries over a 10-year period results in a poorly defined population. An analysis such as LBW is likely to be overpowered with such a large dataset and so may detect statistical differences that are not clinically meaningful. For example, all of the included risk factors had a pvalue<0.2 and were included in the multivariable analysis. For low birthweight, you likely to have enough power in each individual survey and so don’t benefit from this type of pooling. It would be appropriate however to use weighted data from these studies to estimate relative burden of LBW and the risk factors in the larger region or sub-regions. The authors could consider focusing on just one region and stratifying by country and assess for differences by country.

**Author’s Response:-** Thank you very much for your comment. Our objective was to know the pooed magnitude of LBW and factors related to Low Birth Weight (LBW) at the SSA level, not a single region (East, West, Central, and South). Thank you very for your concern related to overfitting. Actually having an adequate sample size gives an efficient estimate and robust standard error. But the problem is overfitting. After your comment, we included this issue in our limitation session(line 300) as it may affect our result. If you know any solution for such types of problems (overfitting) we are ready to learn from you. For this study, we used weighted data for analysis(table 2 weighted frequency done using weighting variable v005).

2) It would be helpful for the authors to provide a flow chart of the data. It would be helpful to know what proportion of respondents were excluded based on missing birthweight and know if the risk factors were similar to those who were included in the study. Please include the number of live births, and what proportion had LBW measured.

**Author’s response:-**Thank you very much for your nice and constructive comments. We accept your comment and included a flow chart of the data. A total of 365,818 live births were recorded in the 35 SSA countries. From this only 202, 878(55%) births were their birth weight measure at birth. 162,940 of them were excluded since their weight was not measured at birth and missingness.

3) Line 101-102: Please provide DHS or other citations (or an appendix) referencing the sampling procedure in each country is the same. It is very likely that the sampling strategies were different in different countries.

**Author’s response:-**Thank you very much for your comment. We accept your comment and we included the citation in the main text. The DHS program uses the same variable code, data collection manual, and sampling procedure though out the world that’s why we appended each country by giving code for each and able to work this analysis. You can read more on (<https://www.dhsprogram.com/pubs/pdf/DHSG1/Guide_to_DHS_Statistics_DHS-7_v2.pdf> ).

4) Line 125: Please provide more details about the meta analysis shown in the forest plot. I assume this was fixed effects meta analysis? Is this how the pooled prevalence was calculated? Please clarify. The forest plot is only a visualization of the analysis.

**Author’s response:-**Thank you very much for your constructive comments**.** We accept your comments and included the meta-analysis part in the method session of data management and analysis section line 156-158 (see the revised manuscript)

5) It seems more levels of the hierarchical model may be needed. Consider clustering on the woman, household, cluster and country (region might not be necessary).

**Author’s Response:-** Thank you very much for your comment. Regarding the levels of a variable actually, some literature uses more than two levels. For our study, we included two levels(individual and community indicated in the independent variable ). We included region (SSA) as **a** community level variable because we believe that there is a variation on LBW in each sub-regions(South, East, West, and Central Africa). The forest plot result supports this.

6) It is not clear how missing data is handled. Was the multivariable model a complete case analysis?

**Author’s Response:-** Thank you very much for your comment. The missing data concerning the outcome(Birth weight(normal vs LBW)) variable was the drop. Not only missing data but also those births with which birth weight not measured at birth were also drop before any statistical analysis (see the revised manuscript in the flow chart of the data).

Results:

7) Line: 166: the text says 202878 mothers were included, yet the variables in table 2 sub to 203,466. Is this due to the weighting? Other numbers in this paragraph also don’t match the tables.

**Author’s Response:-** Thank you very much for your constructive comments. Due to tabulating errors in Stata, some variables were presented without weighing it. We accept your comment and corrected the table figures accordingly after weighing the figure 202,878 is the weighted total sample size figure. (see the revised manuscript).

8) Line 166: I think you mean West Africa and not East Africa?

**Author’s Response:-** Yes that was it was a writing error. Thank you very much for the correction. We accept your comment and corrected it accordingly(see the revised manuscript).

9) Table 2 shows the same number of women and the same number of children. If all children born to women in the last 5 years are included in the analysis, I would expect there to be more children included in the analysis than women included. Please clarify the discrepancy and add the number of women and number of children included in the analysis separately in the text (the flow chart in suggestion 2 would also help this point).

**Author’s Response:-** Thank you very much for your concern related to the number of children and number of women included in this study. If a woman had more than one child five years preceding the survey, the most recent birth was taken because the number of children and women included in this study were the same**.**

10) Sample sizes from table 1 don’t match those listed in figure1

**Author’s Response**:- Thank you very much for your comment. We accept your comment and corrected it accordingly(see the revised manuscript).

11) The variable of the husband’s education is misrepresented. 31904 women who are not married should have a husband’s education as missing.

**Author’s Response:-** Thank you very much for your comment. We accept your comment and corrected it accordingly( see the revised manuscript)

12) Please clarify the meaning of “No” for education status. Do you mean less than primary education?

**Author’s response:-**No education means respondents who had no formal education (can not read and write)

13) Similar to 6: Please check variable for preceding birth interval. How is this defined for a woman’s first birth? If they are in the ≥24 months, this could be the reason for the unexpected results.

**Author’s response:-** This variable very important variable for LBW. Before we fit the model, we exclude women who had one child. as we know stata uses complete case analysis during model fitting. Therefore this issue was resolved before any statistical analysis.

14) Table 4: Please present column % instead of row % for comparison between normal and low birthweight.

**Author’s response:-**Thank you very much for your comment. We accept your comment and corrected it accordingly(see the revised manuscript).

Discussion

15) Overall the discussion could be improved to put the results in context for the reader. Consider restricting the discussion to focus on some key results. The conclusion discusses modifiable risk factors. One approach could be to discuss the modifiable risk factors and the context of interventions for these risk factors. Brining in the discussion of preterm and fetal growth restriction may also bring context to these results.

**Author’s response:-**Thank you very much for your comment. We accept your comment and corrected it accordingly(see the revised manuscript).

16) All statements of explanation for these findings should be based on the literature and so need to be cited (line 227, 233, 237, 240, 249, 252, 254. 258).

**Author’s Response:-** Thank you very much for your comment. We accept your comment and corrected it accordingly(see the revised manuscript).

17)	Line 236: LBW associated with multiples is well established and it is an oversight here not to mention that multiples are almost always born early, and so increased risk for LBW for that reason.

**Author’s Response:-** Thank you very much for your comment. We accept your comment and corrected it accordingly(see the revised manuscript).

**Minor:**

In response to reviewer 3’s suggestions, remove the word determinants in line 38, 184

Introduction:

**Author’s response:-**

1) Line 56: Please define LBW abbreviation – the first sentence could read, Low birth weight (LBW) is one of the….

**Author’s response:- Author’s response:-** Thank you very much for your comment. We accept your comment and corrected it accordingly(see the revised manuscript).

2) Line 61-63: The last sentence in the first paragraph doesn’t seem relevant to this overall summary that is at the SSA level. I suggest deleting or replacing high and low estimates for the region.

**Author’s response:- Author’s response:-** Thank you very much for your comment. We accept your comment and corrected it accordingly(see the revised manuscript).

3)	Line 71 “well facts” should be reworded. Could say are well established.

4)	Line 78 Nutritional shouldn’t be capitalized.

5)	Please provide citations for other LBW studies, meta-analyses systematic reviews referenced in line 83.

6)	89-92. I am not convinced region-level estimates are very informative for interventions and policy decisions.

**Author’s Response:-** Thank you very much for your comment. We accept your comment(3-6) and corrected it accordingly(see the revised manuscript).

Methods:

7)	Line 96-101: Please include the rationale for why these countries were selected. The list does not include the WHO African region or UN Africa region.

**Author’s response**:-WHO had five Africa regions that are (North, West, East, Central, and South). We used the four African regions that represent sub-Saharan Africa (West, East, Central, and South). I hope the data flow char may answer this question(see the revised manuscript).

8)	Line 111: the specific coded values is not necessary to include. This line could just say “LWB was defined as a birth weight <2.5kg, and those ≥2.5kg were considered normal and above normal birthweights.” – this suggestion could be applied throughout the manuscript.

**Author’s response**:-Thank you very much for your comment. We accept your comment(3-6) and corrected it accordingly(see the revised manuscript).

9)	Line 122: “Based on kinds of literature” needs to be edited. It could be revised to say, potential risk factors for LBW were included based on a review of the literature. (Please include a citation).

**Author’s response**:-Thank you very much for your comment. We accept your comment and corrected it accordingly(see the revised manuscript).

10)	Line 156: If the P-value is used to base the decision on inclusion into the multivariable model, please include P-values with the bi-variate results. Because the study is overpowered for these detections, consider lowering the threshold of what is included in the multivariable analysis. If there were variables considered that did not meet this threshold, they should also be included in the manuscript.

**Author’s response**:-Thank you very much for your comment. As we know the sample size is very large and we try to select variables based on literature. As commented previously, this analysis may had a problem of overfitting. We included this as a limitation. We believe that the confidence interval is better than the p-value since it measures precision.

Results:

1)	Line 172: Please reword the description of highest low birth weight and lowest birthweight to make it easier to read. Consider saying highest LBW prevalence, or the lowest rate of LBW births.

**Author’s response**:-Thank you very much for your comment. We accept your comment and corrected it accordingly(see the revised manuscript).

2)	Line 193: Careful saying mothers were less likely to low birthweight. Consider revising to say “were less likely to deliver LBW infants than mothers without formal education:

**Author’s response**:-Thank you very much for your comment. We accept your comment and corrected it accordingly(see the revised manuscript).

3)	Line 201: Type on the confidence interval. Should be 0.91 instead of 0.81 (according to the table)

4)	Line 210: Typo Bing should be being.

5)	Table 2: please define media exposure.

6)	Table 4 CORs are missing on the last page for parity, sex of the child, type of births, iron supplementation.

**Authors’ response:-**Thank you very much for your comment. We accept your comment from 3-6 and corrected it accordingly(see the revised manuscript).

Discussion

7)	Line 229: The statement about Brazil seems irrelevant for a paper focused on SSA- consider removing. The authors could consider comparing to other regions instead.

**Authors’ response:-**Thank you very much for your comment. We accept your comment from 3-6 and corrected it accordingly(see the revised manuscript).

8)	consider the term multiples throughout instead of multiple pregnancies.

**Authors’ response:-**Thank you very much for your comment. We accept your comment from 3-6 and corrected it accordingly(see the revised manuscript).

Considerations

1) It would be helpful to re-orient the risk factors so they are all risk factors or protective factors to help readability.

**Author’s response:-**Thank you very much for your comment. There is only one/two variables change that is a risk almost all are in the protective size. We put the original presentation as it is.

2)	Consider looking for trends in birth weight by year, may want to control for the year of the DHS survey.

**Author’s response:-**Thank you very much for your comment. We only take a single survey for each country the recent one. There to do trend in needs more than one survey for each country that’s not our objective.

3) Consider combining Table 2 and 4.

**Authors’ response:-**Thank you very much for your comment. We accept your comment from 3-6 and corrected it accordingly(see the revised manuscript).
