# Supplementary material for: Prevalence of low birth weight and its associated factor at birth in Sub-Saharan Africa: A generalized linear mixed model
Source: PLoS One. 2021 Mar 11;16(3):e0248417. doi: 10.1371/journal.pone.0248417 (PMC7951905; doi:10.1371/journal.pone.0248417)
Supplement: Attachment — Submitted filename: Point by point response.docx [file pone.0248417.s007.docx]

**PLOS ONE**

**Point by point response for editors/reviewers comments**

The manuscript title “**Prevalence of low birth weight and its associated factor at birth in Sub-Saharan Africa: A Generalized Linear Mixed	Model”**

**Manuscript ID:** **PONE-D-20-27726R3**

**Dear editor/reviewer.**

Dear all,

I would like to thank you for these fair, constructive, building, and improvable comments on this manuscript that would improve the substance and content of the manuscript. We considered each comment and clarification questions of reviewers on the manuscript thoroughly. My point-by-point responses for each comment and question are described in detail on the following pages. Further, the details of changes were shown by track changes in the supplementary document attached.

**Response to Reviewers comments**

**Reviewer #1**

Critical

13) thank you for the explanation about the preceding birth interval variable. The sum of the birth interval variable equals the total sample size (<24=24,427, ≥24=178,450). If you dropped all primigravida women, that means the entire sample size is among multiparous women and this should be clarified in the methods, results, and discussion. If that is not correct, as I suspect the primigravida women are included in one of birth interval categories by mistake. Stata treats a missing variable as a very large number, so depending on how this categorical variable was created those with only one child may be included in the ≥24 category. You may want to include these women in their own category so they can be included in the analysis. Please clarify if this population is among multiparous women or correct the birth interval variable to include primigravida women in their own category.

**Author’s Response:-** Thank you very much for your condtractive and critical issues you raised. We accept you comments corrected accordingly. The reality is that this variables are included in the analysis and we try to explore our data and analysis and we included in the analysis at this stage as separate category. we included the primigravida in the analysis and we corrected in the table (table 3). See the revised manuscript. From a total of 202,878 samples 52,207 of them were primigravida and we include them in the analysis and we recoded this this variable 1-2, 3-5, and 6+ ( see revised table 3 and this number of sample size was edual to birth interval aand method session line 126) (<24=24,427, ≥24=178,450). The following stata output is the sample analysis when explore our data again. Many thanks your careful review of our manuscript. Therefore those population was part of our analysis and we included in the revised session.

Minor:

#12) Thank you for the clarification of No for education. I think it would be helpful to include this in the manuscript for readers that may have the same question.

**Author’s Response:-** Thank you very much for comment and we accept your comment and included this concern in the revised session of our manuscript(see the revised manuscript line number 122-123 ).
